# Utility of opportunistic screening to assess the impact of urinary incontinence on quality of life and barriers to seeking treatment among women attending a tertiary healthcare centre in North India

**DOI:** 10.1186/s12894-024-01434-7

**Published:** 2024-03-02

**Authors:** Priyanka Garg, Lajya Devi Goyal, Suresh Goyal, Madhur Verma

**Affiliations:** 1 Department of Obstetrics and Gynecology, All India Institute of Medical Sciences, Bathinda, Punjab India; 2 Department of Urology, All India Institute of Medical Sciences, Bathinda, Punjab India; 3 Department of Community and Family Medicine, All India Institute of Medical Sciences, Bathinda, Punjab India

**Keywords:** Urinary incontinence, Quality of life, Women’s health, Urogynecology

## Abstract

**Introduction:**

Urinary incontinence (UI) is a common but frequently neglected problem in females, significantly impacting their psychosocial health. The available estimates are an underestimation of a bigger problem. Thus, the study aimed to estimate the prevalence of UI, its associated risk factors, its impact on the Quality of life (QoL), and barriers to treatment-seeking behaviour in women attending tertiary healthcare centres.

**Methods:**

We conducted a cross-sectional study using an opportunistic screening among women visiting a tertiary care hospital in Punjab recruited using multi-stage systematic random sampling. UI was classified as Stress (SUI), Urge (UUI), mixed (MUI), and No Incontinence (UI less than once a week or a month or no complaints) using the International Consultation on Incontinence Questionnaire–Urinary Incontinence Short Form (ICIQ-UI SF). Bivariate analyses were done using the chi-square test to test the association between the dependent and independent variables. The predictors of UI were explored using univariable and multivariable binary logistic regression and depicted using Odds ratio with 95% confidence intervals. The impact of UI on Quality of Life (QoL) was assessed using the Incontinence Impact Questionnaire-Short Form (IIQ-7), and compared among the three UI types using One-Way ANOVA. Treatment barriers were explored using open-ended questions.

**Results:**

Of the 601 women, 19.6% reported UI (stress UI: 10.1%, mixed UI: 6.0%, and urge UI: 3.5%). There were significant clinical-social factors that predicted different types of UI. The UI depicted a significant effect on QoL across all domains of the IIQ-7 (total mean score: 50.8 ± 21.9) compared to women with no incontinence (0.1 + 1.9). The score was highest in women with MUI, followed by SUI and UUI. About two-thirds of the affected women never consulted a doctor and considered it a non-serious condition or a normal ageing process.

**Conclusions:**

The present study found a high prevalence of UI through opportunistic screening across all the women’s age groups with different conditions. Due to the associated stigma, clinicians should make every attempt to talk more about this, especially in women with medical conditions that can precipitate UI. Furthermore, the results call for generating more robust estimates through community-based screening studies.

## Introduction

Urinary incontinence (UI) is a common but frequently neglected problem occurring primarily in females [1]. Defined by the International Continence Society (2002) as an involuntary loss of urine at an inappropriate time and place, UI is one of the main reasons for poor health in women [2]. Worldwide, the estimated prevalence of UI in women is 8.7%, with different studies reporting a prevalence between 23 and 55% [1, 3–5]. In India, the prevalence ranges between 10 and 42%, as per guidelines from the Urological Society of India [2]. The wide variations in the prevalence are attributed to different age groups of participants included in these studies, clinical care conditions, and the definition of incontinence. UI can be categorised as (i) SUI, Stress Urinary Incontinence (Complaint of involuntary loss of urine on effort or physical exertion including sporting activities or on sneezing or coughing), (ii) UUI; Urge urinary incontinence (Complaint of involuntary loss of urine associated with urgency), and (iii) MUI; Mixed urinary incontinence (Complaints of both stress and urgency urinary incontinence, that is, involuntary loss of urine associated with urgency as well as with effort or physical exertion including sporting activities or on sneezing or coughing) [6]. SUI is the most typically reported of the three types, followed by MUI or UUI [7].

The problem is often underreported due to a lack of knowledge or embarrassment in seeking medical advice, especially in rural women. As per the Irish Longitudinal Study on Ageing, underreporting can be as high as 40%, even with frequent symptoms. Despite visiting the doctors for other health problems, women prefer not to disclose UI problems [8]. As a result, such women are prone to skin infections, sexual dysfunction, loss of self-esteem, dependency, depression, increased caregiver burden, and economic cost [9]. Many affected women normalise UI, which ultimately takes a toll on their daily living and impacts their Quality of life (QoL), causing social isolation and restricted lifestyles [10]. Several studies have reported a significant negative impact on the QoL in the affected women [9–11]. The type of UI affects QoL to different degrees. The literature review suggests that the UUI subtype has the highest negative impact on QoL, but other contextual factors—such as age, socioeconomic status, existing medical comorbidities, and the duration of UI symptoms—can have further negative impacts on condition-specific QoL [12]. Therefore, QoL is considered the preferred outcome measure for evaluating UI treatments [13].

While there are numerous studies on UI from developed countries, there are limited data from low-and middle-income countries with a predominantly rural population, such as India, thus making actual disease burden estimates challenging. Moreover, most existing studies are limited to the elderly perimenopausal age groups and have not prioritised a life-cycle approach. Furthermore, few researchers from Punjab have worked towards generating estimates, thus making it essential to study the disease epidemiology within the local context [2]. An updated picture of UI in Indian women will be of great importance in formulating strategies for preventing and controlling UI, reducing the disease burden in the region, and improving their QoL. However, community-based studies are vital for generating evidence around disease prevalence, but UI is difficult to diagnose in field settings and has to rely on self-reported measures. On the other hand, hospital-based studies tend to overestimate the problem due to the inherent bias in selecting study participants. Opportunistic screening is a viable strategy for screening patients as it relies on early disease detection in people who present to healthcare providers with various complaints other than the study variable. Within this background, we conducted this study to estimate the burden of UI, its associated risk factors, its impact on the QoL, and barriers to treatment-seeking behaviour in women attending tertiary healthcare centres through opportunistic screening.

## Methods

We conducted a cross-sectional study between August 2021- March 2022.

### Study settings

The study was conducted in the Outpatient Department (OPD) of a tertiary care centre, AIIMS Bathinda, in the Malwa region of Punjab, India. It is an apex institute with a daily footfall of more than 2000 patients and caters to comprehensive preventive and curative services through its speciality and super-speciality departments. For the purpose of our study, we mention that the institute has functional departments of obstetrics, gynaecology, urology, and neurology, with expertise in managing cases of UI through an interdisciplinary approach on a regular basis.

### Study population

We approached women who were ≥ 18 years of age and were seeking treatment consultations in our institute from any medical or surgical department apart from the urology, obstetrics, and gynaecology departments. We excluded pregnant and lactating women from participating in our study.

### Sample size

The sample size was calculated using an online sample size calculator, openepi version 3.01, available at https://www.openepi.com/SampleSize/SSPropor.htm. A sample size of 246 was calculated using the single population proportion formula after considering the prevalence of UI among women of reproductive age group to be around 20% [14], with a 95% confidence interval, a margin of error of 5%. The sample size was further adjusted with a correction factor 2 (menstruating/menopausal). The total sample size estimate (obtained by summing across the strata) was then adjusted for the design effect (1.2) to get a final sample of 590. It was decided to approach 620 women after considering a non-response rate of 5%.

### Sampling methodology

A multistage systematic random sampling method was used for sample collection. Firstly, two days per week were randomly chosen for sample collection. For the next stage, we disaggregated our study population into different age groups for equal representation of women from different phases of their lives. Then, a systematic random sampling approach was implemented to approach the women, and every fifth woman from the waiting list of different OPDs was invited to participate in the study. The women were duly informed about the purpose of the study and were assured of data confidentiality. Those who agreed to participate in the study were requested to sign the consent form. In case she was illiterate, the participant information sheet was read to her, and her consent was taken in the presence of a witness. Following this, data were collected to assess the study objectives through face-to-face interviews using a pre-tested and pre-validated structured questionnaire. Trained nursing officers conducted the interviews sensitised about the topic and were given the necessary training to evaluate UI and make arrangements for consultation with a gynaecology and urology specialist in case the participants had complaints that were suggestive of UI.

### Study tool

The questionnaire collected information about the study objectives under the following major sections. Section A collected information regarding the demographic and clinical history questionnaire. The variables in this domain included the age of the participants, residence, occupation, marital status, education, menstrual history, obstetrics history, and medical and surgical history. Section B collected information to assess and characterise the type of UI.

### Symptoms severity

The International Consultation on Incontinence Questionnaire–Urinary Incontinence Short Form (ICIQ-UI SF) is highly recommended for evaluating UI symptom severity. The questionnaire helps to evaluate the frequency, severity and impact on quality of life (QoL) of UI in men and women for research and clinical practice. It is a relatively short questionnaire consisting of six items: two demographic items and three items for rating symptoms in the preceding 4-weeks (frequency of UI episodes, the amount of leakage, and overall impact of UI). The total score of these three items gives the ICIQ-UI-SF score with a range of 0 to 21 points, where higher scores indicate greater symptom severity and a higher impact on the women’s QoL. The overall scores can be divided into four severity categories: slight = 1–5 points, moderate = 6–12 points, severe = 13–18 points, and very severe = 19–21 points. It also includes an unrated, self-diagnostic item for assessing the type of UI. UI was classified using standard operational definitions as SUI (symptoms suggestive of leak or loss of urine caused by sneezing, coughing, exercising, lifting, or physical activity); UUI (symptoms suggestive of a sudden urge to void with subsequent leakage before reaching toilet); MUI (symptoms defined as at least one stress and one urge symptom), while No Incontinence was defined when women who reported UI less than once a week or a month or did not report any complaints [15]. The Hindi version of the ICIQ-UI-SF was accessed with permission [16]. The reliability and validity of ICIQ-UI-SF have been reported in previous studies from India [17–19]. UI was recorded if there was a positive history of leakage or involuntary loss of urine during the four weeks before the study.

Further, we assessed the UI severity defined by the frequency of the UI occurrence [9]. As per this, UI was categorised as mild (UI occurring once or twice a week), moderate (once or twice a day), and severe (3 or more times a day). However, we could not consider the amount of UI while determining the severity of UI.

### Impact on QoL

The impact of UI on the QoL of the women was assessed using the Incontinence Impact Questionnaire-Short Form (IIQ-7), a UI-specific psychometric questionnaire that assesses the psychosocial impact of UI in women. It consists of 7 items: 1—Household chores, 2—Physical recreation, 3—Entertainment activities, 4—Travel > 30 min away from home, 5—Social activities, 6—Emotional health (nervousness, depression, etc.), 7—Feeling frustrated; which is subdivided into 4 domains: PA—physical activity (items 1 and 2), TR—travel (items 3 and 4), SA—social activities (item 5), and EH—emotional health (items 6 and 7) [20]. The responses are recorded on a four-point rating scale:, like (i) 0 = not at all, (ii) 1 = slightly, (iii) 2 = moderately, and (iv) 3 = greatly. The score for the scale ranges from 0 to 100, where 0 represents the best condition, while 100 is the worst QoL. This tool was translated and back-translated into Hindi using the standardised World Health Organisation’s translation methodology [21]. The tool has also been previously used in North Indian settings [22].

### Barriers to treatment-seeking behaviour

Once the participants were diagnosed to be living with any UI, they were further enquired about their treatment history. If the patient did not seek treatment, further questioning was done to seek the barriers to treatment using a semi-structured questionnaire. Participants were asked questions like *“Do you consider UI as normal bodily change?”, “If you ever thought UI was a disease, how were you thinking it would get cured ?” “How do you assess the gravity of this condition of having UI?” “What were the reasons you could not seek treatment?” and “Did you ever feel that you should seek treatment but were not able to? If yes, why could you not do that?”*

### Data analysis

The data were entered in MS-EXCEL and analysed using the 21st version of SPSS. Univariate analysis was performed to describe the data and was depicted using frequency and proportions. Bivariate analyses were done using the chi-square test to test the association between the dependent and independent variables. Further, the predictors of UI were explored using univariable binary logistic regression analysis that estimated unadjusted Odds ratios. Significant independent variables with p-value < 0.2 were included to build the final model that finally estimated adjusted odds ratios with 95% Confidence Intervals (aOR; 95%CI) using multivariable binary logistic regression. The aOR > 1 indicates that the independent variable increased the likelihood of having UI and was labelled a risk factor. In contrast, the variables with aOR < 1 decreased the likelihood of having UI and were protective. However, an OR = 1 depicted no association between the independent variables and the likelihood of having UI. Lastly, the responses to questions about QoL were scored appropriately and compared across different categories of UI using one-way ANOVA [23]. A p-value < 0.05 was considered statistically significant.

### Ethical approval

The study was duly approved by the institutional ethical committee of the institute vide letter no. IEC/AIIMS/BTI/120, where this study was conducted. Written informed consent was obtained from all the participants before data collection after the purpose of the study was explained and data confidentiality was ensured.

## Results

We included a total of 601 women in our analysis. Table 1 depicts the study participants’ socio-demographic characteristics with or without UI complaints. It was seen that most of our women who opted in for opportunistic screening were between 30 and 45 years (52.1%), from rural areas (52.7%), were educated (57.7%), overweight or obese (67.9%), and had still not attained their menopause (74.2%). Most of these women were married at < 25 years of age, had one child (66.7%), and delivered vaginally (65.4%). A higher proportion of women had concomitant systemic co-morbidities (15.1%), while a lesser proportion only had gynaecological problems (5.3%). Around 3.5% of women reported complaints suggestive of urinary tract infections.

**Table 1:** Prevalence of Urinary incontinence among the women as per the type and segregated by socio-demographic characteristics

	Sample size	No Incontinence	Stress Urinary Incontinence	Urge Incontinence	Mixed Urinary Incontinence	p-value*
Total	601(100)	483(80.4)	61(10.1)	21(3.5)	36(6.0)	
**Age categories**						< 0.05
< 30 years	95(15.8)	90(94.7)	5(5.3)	0	0	
30–45 years	313(52.1)	273(87.2)	24(7.7)	7(2.2)	9(2.9)	
> 45 years	193(32.1)	120(62.2)	32(16.6)	14(7.3)	27(14)	
**Residence**						< 0.05
Urban	284(47.3)	246(86.6)	26(9.2)	4(1.4)	8(2.8)	
Rural	317(52.7)	237(74.8)	35(11)	17(5.4)	28(8.8)	
**Occupation**						0.131
Working	137(22.8)	117(85.4)	7(5.1)	6(4.4)	7(5.1)	
Non-working	464(77.2)	366(78.9)	54(11.6)	15(3.2)	29(6.3)	
**Education**						< 0.05
Illiterate	254(42.3)	173(68.1)	35(13.8)	19(7.5)	27(10.6)	
Educated	347(57.7)	310(89.3)	26(7.5)	2(0.6)	9(2.6)	
**Overweight/Obese**						< 0.05
No	193(32.1)	179(92.7)	8(4.1)	2(1.0)	4(2.1)	
Yes	408(67.9)	304(74.5)	53(13)	19(4.7)	32(7.8)	
**Menstrual history**						< 0.05
Mensurating	446(74.2)	401(89.9)	27(6.1)	6(1.3)	12(2.7)	
Peri-menopausal	14(2.3)	7(50.0)	4(28.6)	0(0)	3(21.4)	
Menopausal	141(23.5)	75(53.2)	30(21.3)	15(10.6)	21(14.9)	
**Age at marriage**						< 0.05
≤ 25 years	444(73.9)	335(75.5)	56(12.6)	20(4.5)	33(7.4)	
> 25 years	133(22.1)	125(94)	5(3.8)	1(0.8)	2(1.5)	
Unmarried	24(4.0)	23(95.8)	0	0	1(4.2)	
**Parity**						< 0.05
Nulliparous	62(10.3)	60(96.8)	0	0	2(3.2)	
Primiparous	401(66.7)	331(82.5)	36(9.0)	9(2.2)	25(6.2)	
Multiparous	138(23.0)	92(66.7)	25(18.1)	12(8.7)	9(6.5)	
**Mode of delivery**						< 0.05
Not applicable	72(12)	68(94.4)	2(2.8)	0(0)	2(2.8)	
Vaginal	393(65.4)	299(76.1)	45(11.5)	18(4.6)	31(7.9)	
Assisted Vaginal	9(1.5)	3(33.3)	5(55.6)	0	1(11.1)	
Caesarean section	127(21.1)	113(89.0)	9(7.1)	3(2.4)	2(1.6)	
**Associated Comorbidities**						< 0.05
Nil	434(72.2)	391(90.1)	27(6.2)	4(0.9)	12(2.8)	
Only Gynaecological	32(5.3)	19(59.4)	9(28.1)	3(9.4)	1(3.1)	
Systemic	91(15.1)	58(63.7)	12(13.2)	8(8.8)	13(14.3)	
Multi-morbidity	23(3.8)	6(26.1)	6(26.1)	4(17.4)	7(30.4)	
Urinary Tract Infections	21(3.5)	9(42.9)	7(33.3)	2(9.5)	3(14.3)	

*p-value as per Chi-square test

Of the participants, approximately 19.6% reported complaints suggesting UI. Further confirmation was done following detailed history, examination (pelvic examination, Valsalva Maneuver), and necessary investigation (urine routine and microscopy, culture, and sensitivity). It was seen that the majority of these women had SUI (10.1%), followed by MUI (6.0%) and UUI (3.5%) (Table 1). As per different types of UI, we observed significant differences in the distribution of the participants according to their age, with older women > 45 years depicting a higher proportion of SUI and MUI (16.6% and 14%), whereas younger and middle age women showing SUI (5.3, 7.7% respectively). Similarly, there were significant disparities in the proportion of the different types of UI based on their urban-rural residence, education, being overweight/obese, menstrual history, age at marriage, parity, mode of last delivery, and associated comorbidities.

Table 2 explores the risk factors for different types of UI observed in our study. We observed that for SUI, peri-menopausal (aOR: 4.6; 1.1–19.2), menopausal women (aOR: 2.8; 1.7–7.3), marriage at a younger age, vaginal delivery (aOR: 2.2; 1.2–2.7) or through LSCS (aOR: 1.7; 1.3–2.2), and having gynecological comorbidity (aOR: 3.4; 1.3–9.3) or UTI (aOR: 5.5; 1.9–16.3), and being overweight/obese (aOR: 2.5; 1.1–5.7) depicted higher odds of having the SUI. Similarly, UUI was significantly higher in women who were working, illiterate, and menopausal (aOR: 3.3; 1.7–15.5) or were having associated systemic comorbidities (aOR: 5.7; 1.4–23.2), multi-morbidity (aOR: 6.8; 1.1–43.7), or UTI (aOR: 11.9; 1.5–19.3). At the same time, MUI was significantly higher in the presence of multi-morbidities (aOR: 9.0; 2.1–37.5) and UTI (aOR: 7.5; 1.5–37.2).

**Table 2:** Regression analysis to predict risk factors for Urinary incontinence among the women (adjusted Odds ratio with 95% Confidence Interval)

	Stress Urinary Incontinence		Urge Incontinence		Mixed Urinary Incontinence	
	aOR (95% CI)	P-value	aOR (95% CI)	P-value	aOR (95% CI)	P-value
**Age categories**						
< 30 years	Ref		Ref		Ref	
30–45 years	0.7(0.2–2.6)	> 0.05	1.2(0.7–1.9)	> 0.05	1.3(0.8–1.7)	> 0.05
> 45 years	0.7(0.2–2.2)	> 0.05	1.3(0.9–1.7)	> 0.05	1.7(0.7–1.9)	> 0.05
**Marital Status**						
Not in union	Ref		Ref		Ref	
In union	2.7(0.7–10.4)	> 0.05	0(0-0.1)	> 0.05	0.7(0.1–4.2)	> 0.05
**Residence**						
Urban	Ref		Ref		Ref	
Rural	0.5(0.3–1.1)	> 0.05	1.4(0.4–5.5)	> 0.05	2.0(0.7–5.4)	> 0.05
**Occupation**						
Working	Ref		Ref		Ref	
Non-working	1.5(0.6–3.8)	0.4	0.1(0-0.4)	< 0.05	0.4(0.1–1.2)	> 0.05
**Education**						
Illiterate	Ref		Ref		Ref	
Educated	0.9(0.4–1.9)	0.8	0.2(0-0.9)	< 0.05	0.6(0.2–1.8)	> 0.05
**Total Family Income**						
≤ 10,000 ₹	Ref		Ref		Ref	
> 10,000 ₹	1.4(0.7–2.5)	0.3	0.4(0.1–1.5)	0.2	1.1(0.5–2.5)	> 0.05
**Menstrual history**						
Mensurating	Ref		Ref		Ref	
Peri-menopausal	4.6(1.1–19.2)	< 0.05	0.7(0.3-1)	> 0.05	2.3(0.4–11.6)	> 0.05
Menopausal	2.8(1.7–7.3)	> 0.05	3.3(1.7–15.5)	< 0.05	2.2(0.7–6.6)	> 0.05
**Age at marriage**						
≤ 25 years	Ref		Ref		Ref	
> 25 years	0.3(0.1–0.9)	< 0.05	0.3(0-3.3)	0.3	0.4(0.1-2.0)	> 0.05
Unmarried	0.4(0.8-1)	> 0.05	1.5(0.7–1.8)	> 0.05	3.9(0.3–3.3)	> 0.05
**Mode of delivery**						
Not applicable	Ref		Ref		Ref	
Vaginal	2.2(1.2–2.7)	< 0.05	7.1(0.6–8.8)	> 0.05	4.3(0.2–6.6)	> 0.05
Assisted Vaginal	0.5(0.1–5.3)	0.6	0	1	1.2(0.3–1.4)	> 0.05
Cesarean section	1.7(1.3–2.2)	< 0.05	3.6(0.1–5.7)	> 0.05	4.7(0.2–7.5)	> 0.05
**Associated Comorbidities**						
Nil	Ref		Ref		Ref	
Only Gynaecological	3.4(1.3–9.3)	< 0.05	4.3(0.7–25.4)	> 0.05	0.3(0.0-3.1)	> 0.05
Systemic	1.1(0.5–2.6)	0.8	5.7(1.4–23.2)	< 0.05	1.9(0.7-5.0)	> 0.05
Multi-morbidity	1.1(0.3-4)	0.9	6.8(1.1–43.7)	< 0.05	9.0(2.1–37.5)	< 0.05
Urinary Tract Infections	5.5(1.9–16.3)	< 0.05	11.9(1.5–19.3)	< 0.05	7.5(1.5–37.2)	< 0.05
**Overweight/Obese**						
No	Ref		Ref		Ref	
Yes	2.5(1.1–5.7)	< 0.05	3.6(0.7–17.9)	> 0.05	2.6(0.8–8.3)	> 0.05

UI depicted a significant effect on QoL across all domains of the IIQ-7 compared to women with no symptoms suggestive of UI **(**Table 3**)**. The total mean score in the women with UI was 50.8 ± 21.9, significantly higher than those without UI. The score was highest in women with MUI, followed by SUI and UUI. Minimum mean scores were depicted for the entertainment activities in the travel domain and the ability to do household chores in the physical activity domain. In contrast, the emotional health domain had the highest scores under the feeling frustrating item.

Of the 118 (19.6%) women who were confirmed to have any UI, two-fifths (8.0%; *n* = 48) ever consulted a doctor, while nearly three-fifths (11.6%; *n* = 70) never did so **(**Table 4**)**. The most commonly cited reason for not seeking help included non-serious perceptions about the condition (6.0%), followed by reasons like “will get cured naturally” (3.0%), shyness (1.7%), and some considered it to be a normal ageing process (0.7%).

**Table 3:** Effect of urinary incontinence subtypes on the quality of life among female participants included in the study (mean ± SD)

Items in different domains of Incontinence Impact Questionnaire-Short Form (IIQ-7)	No Incontinence	Urinary incontinence (any type)	Stress Urinary Incontinence	Urge Incontinence	Mixed Urinary Incontinence	p-value*
	*N* = 483	*N* = 118	*N* = 61	*N* = 21	*N* = 36	
**Physical activity**						
Ability to do household chores (cooking, housecleaning, laundry)?	0	0.3 ± 0.6	1.1 ± 0.8	1.1 ± 0.8	1.7 ± 0.8	< 0.001
Physical recreation such as walking, swimming, or other exercise?	0	0.3 ± 0.7	1.3 ± 0.7	1.2 ± 1	1.7 ± 0.8	< 0.001
**Travel**						
Entertaining activities (movies, concerts, etc.)?	0	0.2 ± 0.6	1.2 ± 0.8	1.2 ± 0.9	1.4 ± 0.9	< 0.001
Ability to travel by car or bus more than 30 min from home?	0	0.3 ± 0.7	1.5 ± 0.7	1.4 ± 1.0	1.9 ± 0.8	< 0.001
**Social/relationships**						
Participation in social activities outside your home?	0	0.3 ± 0.7	1.5 ± 0.9	1.4 ± 1.0	1.9 ± 0.8	< 0.001
**Emotional health**						
Emotional health (nervousness, depression, etc.)?	0 + 0.1	0.3 ± 0.8	1.5 ± 1.0	1.4 ± 1.0	2.0 ± 0.8	< 0.001
Feeling frustrated?	0 + 0.1	0.4 ± 0.8	1.6 ± 0.9	1.6 ± 1.1	2.1 ± 0.6	< 0.001
**Total score**	0.1 + 1.9	50.8 ± 21.9	45.8 ± 21	44.9 ± 26.9	60.1 + 17.1	< 0.001

*p-value as per One-Way ANOVA

**Table 4:** Barriers to seeking health care services for managing urinary incontinence among women participants included in the study (*N* = 601)

	Counts	% from overall sample	% within UI group
Participants with any kind of incontinence	**118**	**19.6**	**100**
**Ever consulted a doctor**			
Yes	48	8.0	40.6
No	70	11.6	59.3
**Reasons for not seeking care**			
Did not take it seriously	36	6.0	30.5
Will get cured naturally	18	3.0	15.3
Shyness.	10	1.7	8.5
Considered it as normal aging process	4	0.7	3.4
Any other	1	0.2	0.8
No one to accompany	1	0.2	0.8

## Discussion

UI is a debilitating condition affecting women of all age groups. Most of the previous studies from India have focussed on assessing the burden of UI and its effects on one or the other life stages of women, failing to provide a comprehensive picture. To the best of our knowledge, this is amongst the first studies from India with the potential to fill in this void. We assessed our objectives using an opportunistic screening approach, as community-based studies face difficulty generating reliable estimates. Our screening process was confirmed by a protocol-based evaluation and diagnostic process, which makes our estimates comparatively robust. Key findings are emerging from our study. First, opportunistic screening depicted a high burden, and about one in five women had complaints suggesting any UI. Second, the type of UI significantly varied by age, education, and women-specific characteristics. SUI was predicted by menstrual status, early age at marriage, type of delivery, UTI, and obesity. The likelihood of having UUI was significantly affected by working and education status and was higher in menopausal women with comorbidities, including obesity. While only the presence of multi-morbidity or UTI predicted MUI. Third, UI significantly affected the QoL in the affected women. Fourth, one-fifth of the affected women showed poor treatment-seeking behavior for various reasons.

The prevalence of UI in women visiting the OPD per hospital-based opportunistic screening observed in our study was around 19.6%. Of these, SUI was the most typical type seen at 10.1%, followed by MUI at 6% and UUI at 3.5%. This is similar to the study conducted by Singh et al., who reported an overall prevalence of 21.8%, among whom SUI was the most frequent (16.1%), followed by MUI (3.6%) and UUI (2.07%) [14]. **A**nother hospital-based study from Kerala reported consistent findings of the prevalence of UI to be 26.47%, with SUI being the most frequent (SUI:13.9%, MUI: 7.2%, and UUI 5.4%) [24]. To give a perspective, this prevalence is higher than the reported prevalence among women > 45 years from Punjab as a part of a national-level survey- the Longitudinal Aging Study in India [25]. However, the prevalence reported in LASI is self-reported and may be an underestimate. Because it has been seen that women may not even see this as a problem that requires management, leave apart reporting. A cross-sectional study in Gujrat by Charpot V et al. reported the prevalence of UI around 29% among women in the reproductive age group [26]. In another community-based study, the prevalence has been reported to be even higher in the same age group of women (46.3%) [27]. Other hospital or community-based assessment studies generated estimates that may range between 5 and 70% [3]. However, few studies found MUI the most frequent type. This wide variation in the disease burden is attributed to various definitions used and different study populations and types of methodology. Several factors have been elaborated for UI in post-menopausal women, but UI in young females is less discussed. It has been linked to lifestyle factors like caffeine or alcohol, increased sedentary life leading to sarcopenia, constipation, dietary deficiency including B12, and hormonal fluctuations attributed to different phases of the menstrual cycle [28, 29]. Some hypotheses also link hormonal fluctuations attributed to oral contraceptive pills as an important reason for UI in young females [30].

UI is an important but often neglected health problem associated with modifiable and non-modifiable risk factors. Identifying and altering these risk factors can help reduce the burden and related morbidity. In our study, we found that the prevalence of UI increased, with age being highest (61.8%) in women aged > 45 years (*p* < 0.05). This is in coherence with a study by Ganapathy T showing that the prevalence was high (59.7%) in women > 40 years of age [31]. Singh et al. also reported that the prevalence of UI increased with advancing age (27.8–42.8%) [14]. Congruent findings were seen in other studies in India and outside [4, 9, 11]. This can be explained by the age-related weakening of pelvic floor muscles, reduced contractility, and altered hormonal milieu, i.e., decreased estrogen [32].

Our study depicted that UI occurs more frequently in uneducated women and those residing in rural areas, which was statistically significant (*p* < 0.05). This result is consistent with other studies proving an inverse relationship between the literacy level of women and the prevalence of UI [9]. This could be because uneducated women accept UI as normal and lack knowledge about the disease. Also, these women, especially those residing in rural areas, perceive UI as embarrassing and shameful. Using the bivariate analysis, we observed a higher prevalence of SUI in young women from urban areas. In comparison, women of older age from rural areas depicted a higher prevalence of the MUI. SUI in young urban females is attributed to heavy workload in urban areas, without adequate rest. Although physical activity strengthens pelvic floor muscles, overstretching and overstraining can cause the opposite effect [26]. The high prevalence in rural areas might be because of more engagement in manual labor than in urban areas. Manual work is more likely to increase abdominal pressure and cause damage to pelvic floor muscles and ligaments, thereby increasing the prevalence of SUI [33]. The high burden among women coming from rural areas may also be attributed to higher parity and deliveries conducted by less trained health workers that affect the strength of pelvic floor muscles, contributing to UI in later stages of life.

We also observed that the prevalence was higher in women who were obese, and body mass index (BMI) was a significant predictor of UI. A previous systematic review has also depicted an apparent dose-response effect of weight on urinary incontinence, with each 5-unit increase in BMI associated with about a 20–70% increase in the risk of developing UI risk [34]. It is theorized that excess BMI accentuates the intra-abdominal pressure, which is responsible for increased bladder pressure and urethral mobility, leading to SUI and intensifying the detrusor instability and overactive bladder. This is very similar to pregnancy, where increased body fat causes chronic strain, stretching, and weakening of the normal anatomy of the pelvic floor [35]. SUI was the most common among all the stages of menstruation in our study participants. The American College of Obstetricians and Gynecologists (ACOG) patient information on UI does not list menstruation or ovulation as a source for either UUI or SUI [36]. Apart from some reasons for UI cited above, other issues may include neuromuscular disorders, a pelvic floor disorder, including pelvic organ prolapse and sometimes even bowel leakage, and problems with the actual anatomical structures, including bladder stones. In the current study, early marriage and higher parity were significant predictors of UI. Previous studies have also cited that early sexual initiation and increased childbirths are associated with a higher risk of developing UI [37, 38]. Likewise, in our study, the prevalence of UI was highest among women with normal vaginal delivery (80%) compared to those who had undergone a lower-segment cesarean Sect. (11.8%) or nulliparous women (0.03%). Childbearing is an established risk factor for urinary incontinence among young and middle-aged women. It has been suggested that vaginal delivery is the main contributing factor, possibly because of damage to pelvic floor muscle tissue and nerves [39]. However, pregnancy may cause mechanical and hormonal changes, leading to urinary incontinence. SUI depicted the most significant association with vaginal delivery, which agrees with other studies [14]. Our results agree with previous studies, but this should not be used to justify increased cesarean Sects. [14, 32, 39]. Also, results from the Norwegian EPINCONT study (EPidemiology of INCOntinence in the county of Nord-Trøndelag) suggest that parity is a significant risk factor for UI in early age groups is significantly associated with SUI and MUI [40].

A significant association was seen between UI and the presence of other co-morbidities like diabetes and hypertension. This contrasts with the results from a previous hospital-based study [14]. As per biological plausibility, UI in T2DM can be attributed to microvascular complications and is mainly associated with UUI. It is pertinent to mention here that UUI caused by microvascular damage leads to alterations in detrusor muscle function, innervation, and function of the neuronal component. In contrast, SUI is due to dysfunction of the striated muscle of the urethral sphincter and pelvic floor muscles and their innervations [41]. However, UI in hypertension is mainly seen as a side effect of medication that controls high blood pressure. Most commonly used drugs work by dilating blood vessels but can also interfere with the bladder’s ability to contract or due to the direct effect of diuretics, leading to UI. All these symptoms are exacerbated as hypertensives are overweight and obese, leading to weakened pelvic floors due to sarcopenia [42, 43].

Our study showed that QoL in women with UI was much worse than those without UI, similar to results from previous studies [44, 45]. All the domains of life, including physical activity, travel, social/relationships, and emotional health, were negatively affected. Even though UI significantly affects the QoL, women do not seek treatment for the same. In the current study, almost 60% of the women did not consult anyone for their UI. They mentioned several reasons for non-consultation. More than half of the women (51.4%) considered UI a non-serious problem. A similar rationale was cited in other studies also [46]. Indian women have an immense tolerance threshold for their health.

The major strength lies in the inclusivity of the study. While most of the previous studies focussed on particular conditions or stages of life, the present study included participants from different phases of their lives with different conditions, thus helping us understand the broader implications of this condition. Certain limitations of this study should also be acknowledged. Firstly, being a hospital-based study, the generated prevalence rates may be overestimated. However, looking at the difficulty in confirming the diagnosis through community-based surveys and opportunistic screening, it was the feasible approach for the study, as gynecologists and urologists confirmed the diagnosis and promptly managed it. While the confirmation of diagnosis by the clinicians minimises information bias that may be present in the case of self-reporting, the chances of selection bias in opportunistic screening cannot be ruled out, which can affect the generalizability of the findings to the broader population. Unlike other clinical conditions, there is still a dearth of well-established predictors (3P- polydipsia, polyuria, polyphagia in Type 2 DM) that can prompt the screening for UI; hence, there is always a scope of selection bias in our study, though we tried to make the selection process as reproducible as possible. Cause and effect relationships could not be established due to the study’s cross-sectional nature. All variables were based on self-reporting and could not be confirmed by any diagnostic methods.

In conclusion, the present study found a high burden of UI through opportunistic screening of women in different stages of life. One in five women may be living with UI of any type. Several factors were significantly associated with UI in our study participants, including socio-demographic disparities, menopausal status, age of marriage, parity, type of delivery, and comorbidities like DM, hypertension, and obesity. We also observed a significant impact of UI on QoL. Despite the high burden, less than half of the affected women ever consulted a doctor for UI, due to different barriers. Hence, clinicians should make every attempt to talk more about UI, especially in women with high-risk factors that can precipitate UI. The study highlights the need to raise awareness around UI, as patients usually do not seek treatment and suffer alone. Further studies can suggest measures to dispel the myth that UI is normal or that it cures on its own. The present study adds data to the mounting evidence that factors associated with UI are similar in other populations and need close attention in clinical practice. Furthermore, the results call for generating more robust estimates through community-based screening studies and highlight the importance of interventions that reduce modifiable factors.

## Data Availability

The dataset is available with the lead author, Priyanka Garg, and it can be accessed upon reasonable request.
